# The most important variables associated with death due to COVID‐19 disease, based on three data mining models Decision Tree, AdaBoost, and Support Vector Machine: A cross‐sectional study

**DOI:** 10.1002/hsr2.2266

**Published:** 2024-07-25

**Authors:** Bita Shokri gharehhasani, Mansour Rezaei, Armin Naghipour, Nazanine Sayad, Shayan Mostafaei, Ehsan Alimohammadi

**Affiliations:** ^1^ Taleghani Hospital Kermanshah University of Medical Sciences Kermanshah Iran; ^2^ Social Development and Health Promotion Research Center Kermanshah University of Medical Sciences Kermanshah Iran; ^3^ Clinical Research Development Center, Imam Khomeini and Dr.Mohammad Kermanshahi and Farabi Hospitals Kermanshah University of Medical Sciences Kermanshah Iran; ^4^ Kermanshah University of Medical Sciences Kermanshah Iran; ^5^ Department of Medical Epidemiology and Biostatistics Karolinska Institute Stockholm Sweden; ^6^ Department of Neurosurgery Kermanshah University of Medical Sciences Kermanshah Iran

**Keywords:** AdaBoost, COVID‐19, data mining models, death, Decision Tree, effective factors, Support Vector Machine

## Abstract

**Introduction:**

Death due to covid‐19 is one of the biggest health challenges in the world. There are many models that can predict death due to COVID‐19. This study aimed to fit and compare Decision Tree (DT), Support Vector Machine (SVM), and AdaBoost models to predict death due to COVID‐19.

**Methods:**

To describe the variables, mean (SD) and frequency (%) were reported. To determine the relationship between the variables and the death caused by COVID‐19, chi‐square test was performed with a significance level of 0.05. To compare DT, SVM and AdaBoost models for predicting death due to COVID‐19 from sensitivity, specificity, accuracy and the area under the rock curve under R software using psych, caTools, random over‐sampling examples, rpart, rpartplot packages was done.

**Results:**

Out of the total of 23,054 patients studied, 10,935 cases (46.5%) were women, and 12,569 cases (53.5%) were men. Additionally, the mean age of the patients was 54.9 ± 21.0 years. There is a statistically significant relationship between gender, fever, cough, muscle pain, smell and taste, abdominal pain, nausea and vomiting, diarrhea, anorexia, dizziness, chest pain, intubation, cancer, diabetes, chronic blood disease, Violation of immunity, pregnancy, Dialysis, chronic lung disease with the death of covid‐19 patients showed (*p* < 0.05). The results showed that the sensitivity, specificity, accuracy and the area under the receiver operating characteristic curve were respectively 0.60, 0.68, 0.71, and 0.75 in the DT model, 0.54, 0.62, 0.63, and 0.71 in the SVM model, and 0.59, 0.65, 0.69 and 0.74 in the AdaBoost model.

**Conclusion:**

The results showed that DT had a high predictive power compared to other data mining models. Therefore, it is suggested to researchers in different fields to use DT to predict the studied variables. Also, it is suggested to use other approaches such as random forest or XGBoost to improve the accuracy in future studies.

## 1 INTRODUCTION

In December 2019, a pneumonia prevalence of unknown origin was reported in Wuhan city, Hubei province, China.^1^ Pneumonia cases were epidemiologically linked to the Huanan seafood wholesale market. Inoculation of respiratory samples into human airway epithelial cells, the Vero E6 and Huh7 cell lines, led to the isolation of a novel respiratory virus, which genome analysis showed to be a novel SARS‐Cov related coronavirus and therefore, it was named as acute respiratory syndrome of the coronavirus (SARS‐CoV‐2) which is a beta coronavirus belonging to the Sarbeco virus subgenus.^2^ The global spread of SARS‐CoV‐2 and thousands of deaths from the coronavirus disease (COVID‐19) led the World Health Organization to declare the disease a pandemic on March 12, 2020. To this date, the world has taken a heavy toll (including human toll, economic consequences, and increased poverty) in this pandemic.^3^, ^4^ The family of coronaviruses in humans, mammals and birds have been identified in terms of genotyping and serology with four types of alpha, beta, gamma and delta which causes disease in humans by alpha and beta species.^5^ As of January 17, 2020, 62 cases of this coronavirus have been confirmed in China and three cases outside of China (2 cases in Thailand and 1 case in Japan).^6^ This new virus with its unknown nature and high prevalence involved the whole world after a short time.^7^ The common symptoms of this disease are: fever, cough, shortness of breath, sore throat, myalgia, and rhinorrhea and the important factors of that are age, cardiovascular diseases and chronic lung diseases.^8^ The results of a systematic review study showed that the elderly, men, blacks, obese people, smokers, diabetics, cardiovascular patients, kidney disease, and hypertension are more at risk of hospitalization due to COVID‐19 infection, which can be considered by relevant experts.^9^ The remarkable thing about patients in the acute phase of this disease is that people suffer from severe complications that may affect their lives for years.^10^ The coronavirus causes various diseases, including respiratory, intestinal, kidney, heart, and nervous diseases.^11^ Also, a study has determined that the social, personal, economic and health effects of this disease may remain in societies for many years.^12^, ^13^

Studies have shown that data mining models, including DT, can be useful for modeling and predicting people at risk of COVID‐19.^5^ Many studies have been conducted to identify the most important variables related to various diseases, in which data mining models have been used and the correctness of these models has been confirmed.^14^, ^15^ Studies have also investigated the most important factors related to death in patients with COVID‐19 using data mining algorithms. There are differences in the effect or importance of factors related to death in these patients in many countries, therefore, it is possible to evaluate the role of environmental, geographical, racial, cultural, and nutritional factors in the mortality rate of patients in different places.^16^ The DT has always been of interest due to its simplicity in fitting and the high speed of execution and the lack of a very high sample size and a specific hypothesis. Also, the SVM algorithm often has a better performance compared to the DT, and in situations where the data is hyperdimensional or the classes are not very clean and separable, using a kernel function has a relatively good performance. However, the performance of the SVM depends on the type of kernel function and the existence of noisy data, and the need for time and sample size is relatively high. On the other hand, Adaptive Boosting (AdaBoost) algorithm is proposed as a basic classifier due to the possibility of training for nonlinear communication, high speed in fitting, and the possibility of using multiple classifiers (such as DT, random forest, and SVM) and with Using the combined technique of Booting is effective in reducing skewness. In a study that was conducted with the aim of comparing data mining models in diagnosing factors related to diseases, the DT performed better than other models.^15^ In another study conducted for the prognosis of cervical cancer, the AdaBoost algorithm was more accurate than the genetic algorithm in cervical cancer prognosis classifications.^17^, ^18^ In other studies, the AdaBoost algorithm has been introduced as a strong algorithm in the classification of variables.^18^, ^19^, ^20^, ^21^

It should be noted that there is no absolute “best” among different algorithms and statistical models, and the accuracy of different models in data classification depends on the nature of the data too. Therefore, the model should be selected according to the desired data and performance. According to the existence of different methods for classifying data, to achieve the optimal model, the comparison of models should be done. To the best of our knowledge, no study has yet been undertaken to concurrently identify the most significant variables associated with COVID‐19‐related mortality in Kermanshah, Western Iran, utilizing the three data mining models (DT, SVM, and AdaBoost). Considering the importance of identifying the most important factors related to death in COVID‐19 patients and to take appropriate measures to reduce the mortality rate in these patients, comparing these three data mining models and determining their sensitivity and specificity to identify the most important factors related to death, we have discussed the disease of COVID‐19 in the city of Kermanshah, Iran.

## 2 MATERIALS AND METHODS

### 2.1 Type and scope of study

The current cross‐sectional study was conducted in Kermanshah hospitals from February 20, 2020, to February 19, 2021. Kermanshah is one of the western provinces of Iran and has a population of about two million people (61.7% urban residents, 37.7% rural residents and the rest nonresidents). It is the capital of the province and its dominant ethnicity is Kurdish.^22^

### 2.2 Sample and methods of collection information

The samples were selected by census method. The sample included outpatients and in patients in Imam Reza, Farabi, Golestan and Shohada hospitals in Kermanshah‐Iran. In the initial review, the information of 50,000 people was registered in the HIS system. But a number of these repeated people and the other number of polymerase chain reaction (PCR) results (PCR was the most reliable test at the time of Covid‐19^23^) were excluded from the study. After checking and cleaning, we reached the sample size of 23,054 people.

### 2.3 Data mining models

In this study, three data mining models Support Vector Machine (SVM), DT, and AdaBoost were used. DT model is one of the most powerful and widely used data mining algorithms for prediction. Among the reasons for the popularity of this model: clarity, comprehensibility, flexibility and relatively fast processing. In this study, three data mining models SVM, DT, and AdaBoost were used. These models are among the most powerful and widely used data mining algorithms for prediction.^18^, ^24^ Among the reasons for their popularity are clarity, comprehensibility, flexibility and relatively fast processing. In this study, the observer method was used to fit the models. For this purpose, 70% of the data were divided for training and 30% for testing (70% training data set, 30% test data set).

### 2.4 Statistical analysis

To describe the studied variables, the frequency (percentage) of reporting. Chi‐square test was used to determine the relationship between independent variables and patients' status (discharged, died). A significance level of 0.05 was considered. Then, the criteria of accuracy, sensitivity, specificity and area under the rock curve were used to compare the predictive power of three data mining models: SVM, DT, and AdaBoost. Data analysis was done under R version 3.5.1 software. To evaluate the models, data mining was done from packages psych, caTools, random over‐sampling examples, rpart, rpartplot, and adaboost under this software.

## 3 RESULTS

Out of the total of 23,054 patients studied, 10,935 cases (46.5%) were women, and 12,569 cases (53.5%) were men. Additionally, the average age of the patients was 54.9 ± 21.0 years. Among these individuals, 22,842 (97.2%) were residents of Kermanshah city, while 662 (2.8%) came from other cities/county. The nationality of 159(0.5%) people was foreign, 100 (0.4%) people were of unknown nationality, and the remaining 22,795 (99.0%) were Iranian citizens. Out of these, 3298 cases required admission to the intensive care unit. Patients had four methods of hospital access available: personal referral, private ambulance, medical center ambulances, or by calling the EMS center at 115. Specifically, 148 individuals (0.6%) utilized private ambulances, 513 people (2.2%) used the 115 center ambulances, 1,333 individuals (7.6%) relied on health center ambulances, and 21,060 people (89.6%) were referred to the health center by personal means. Comparing two groups, one with the outcome event and the other without, revealed a significantly higher mortality rate among men (57.5%) compared to women (42.5%) (*p* = 0.001). Additionally, patients residing in Kermanshah city (97.7%) experienced a higher incidence of mortality outcomes than those in other cities/county (2.3%) (Table 1).

**Table 1:** Demographic characteristics of COVID‐19 patients based on vital status.

Variables	Level	Total (*n* = 23,504)	Patient Alive (*n* = 21,434, 91.2%)	Patient Died (*n* = 2070, 8.8%)	*p*
Gender	Man	12,569 (53.5)	11,379 (53.1)	1190 (57.5)	0.001
	Female	10,935 (46.5)	10,055 (46.9)	880 (42.5)	
Residence	Kermanshah	22,842 (97.2)	20,820 (97.1)	2022 (97.7)	0.152
	Other cities/county	662 (2.8)	614 (2.9)	48 (2.3)	
Nationality	Iranian	22,795 (99.1)	20,725 (89.9)	2070 (9.1)	0.159
	Foreign	159 (0.5)	159 (0.7)	0 (0.0)	
	Unknown	100 (0.4)	100 (0.4)	0 (0.0)	
Place of hospitalization	ICU	3298 (14.9)	2693 (14.5)	605 (22.9)	0.001
	Wards	20,206 (85.0)	18,164 (85.5)	2042 (77.1)	
How to refer	Private ambulance	148 (0.6)	138 (0.6)	10 (0.5)	<0.001
	Med. centers ambulance	513 (2.2)	443 (2.1)	70 (3.4)	
	Contact the EMS or 115	1333 (7.6)	973 (6.6)	360 (17.4)	
	Personal refer	21,060 (89.6)	19,430 (90.7)	1630 (78.7)	
Contact history	Having	10,093 (42.9)	9108 (42.5)	985 (47.6)	0.001
	Not having	13,411 (57.1)	12,326 (57.5)	1085 (52.4)	

*Note*: *p*: *p* value from chi‐square test; Percentages were calculated on the basis of participants with available data.

Abbreviation: EMS, Emergency Medical Service.

In this study, out of the 23480 patients included, 2070 patients (8.8%) experienced mortality. The results indicate a range of clinical findings: severe fever was observed in 6964 cases (29.6%), frequent cough was reported in 9171 cases (39.0%), severe muscle pain was noted in 5697 cases (24.2%), stress and mental distress were prevalent in 13,425 cases (57.1%), low levels of consciousness were detected in 1060 cases (4.5%), loss of the sense of smell affected 369 cases (1.5%), loss of the sense of taste was experienced by 1080 cases (4.6%), abdominal pain was reported in 697 cases (2.9%), nausea was present in 1080 cases (4.6%), vomiting occurred in 1063 cases (4.5%), diarrhea was observed in 663 cases (2.8%), unprecedented anorexia was noted in 1536 cases (6.5%), severe headache affected 1297 cases (5.5%), dizziness was reported in 428 cases (1.8%), chest pain was experienced by 872 cases (3.7%), intubation, necessitating artificial respiration, was required for 1758 cases (7.5%), 528 cases (2.3%) were smokers, 440 cases (1.9%) had a history of drug addiction, 471 people (2.0%) had various types of cancer, with 97 people (0.4%) undergoing chemotherapy, diabetes was diagnosed in 1813 cases (7.7%), chronic blood diseases were present in 105 cases (0.5%), 14 cases (0.1%) were diagnosed with AIDS, immune system deficiencies were observed in 40 cases (0.2%), 256 cases (1.1%) were pregnant, 238 cases (1.0%) were undergoing dialysis, asthma affected 292 (1.2%) patients, and chronic lung disease was reported in 445 cases (1.9%) (Table 2). The most common signs and clinical indicators in patients who died were stress and mental distress (70.4%), frequent cough (29.8%), intubation (37.7%), and chemotherapy (53.1%), respectively (Table 2).

**Table 2:** Comparison of clinical indicators based on vital status (alive or died).

Variables	Level	Patient alive	Patient died	*p*
Habits	Cigarettes	476 (2.2)	52 (2.5)	0.393
	Drug addiction	392 (1.8)	48 (2.3)	0.116
Vital Signs	Fever	6526 (30.4)	438 (21.2)	<0.001
	Frequent coughs	8554 (39.9)	617 (29.8)	<0.001
	Severe muscle pains	5285 (24.7)	412 (19.9)	<0.001
	Stress and mental distress	11968 (55.8)	1457 (70.4)	<0.001
	Low level of consciousness	745 (3.5)	315 (15.2)	0.001
	Loss of smell	352 (1.6)	17 (0.8)	0.004
	Loss of taste	299 (1.4)	24 (1.2)	0.009
	Abdominal pain	667 (3.1)	30 (1.4)	<0.001
	Nausea	1017 (5.0)	63 (3.1)	<0.001
	Vomit	1015 (5.0)	48 (2.4)	<0.001
	Diarrhea	636 (3.0)	27 (1.3)	<0.001
	Unprecedented anorexia	1395 (6.8)	141 (6.9)	0.001
	Unprecedented headache	1246 (5.8)	51 (2.5)	0.087
	Dizziness	400 (2.0)	28 (1.4)	0.001
	Chest pain	806 (4.0)	66 (3.2)	0.007
	Intubation	977 (4.6)	781 (37.7)	0.001
Suffering from diseases	Various types of cancer	407 (1.9)	64 (3.1)	0.001
	Chemotherapy	80 (50)	17 (53.1)	0.047
	Diabetes	1595 (7.4)	218 (10.5)	0.001
	Chronic blood disease	96 (0.4)	9 (0.4)	0.032
	AIDS	13 (0.1)	1 (0.00)	0.826
	Defects in the body's immune system	38 (0.2)	2 (0.1)	0.005
	Pregnancy	237 (1.1)	19 (1.0)	0.001
	Dialysis	218 ((0.8)	20 (1.0)	0.001
	Asthma	272 (1.3)	20 (1.0)	0.235
	Chronic lung disease	393 (1.8)	52 (2.5)	0.031

*Note*: *p*: *p* Value from chi‐square test; Percentages were calculated on the basis of participants with available data.

Abbreviation: AIDS, Acquired Immune Deficiency Syndrome.

Among the three reviewed algorithms, when implemented on the data set, the two algorithms AdaBoost, and DT presented a more reasonable feature selection. In the AdaBoost model, the variables of age, chronic blood disease, fever, cough, muscle pain, immune system deficiency, low level of consciousness, diarrhea, and vomiting were selected in order of importance. In the DT model, the variables of age, fever, cough, muscle pain, sense of taste, abdominal pain, vomiting, diarrhea, chronic blood disease, chronic lung disease, and neurological disorders were selected in order of importance.

The models used in this study were compared in terms of accuracy, sensitivity, specificity and ROC curve level (Table 3).

**Table 3:** Comparison of models by evaluation indices.

Indices	DT	AdaBoost	SVM
Accuracy	0.75	0.74	0.71
Sensitivity	0.60	0.59	0.54
Specificity	0.68	0.65	0.62
Area under the ROC curve	0.71	0.69	0.63

Abbreviations: DT, Decision Tree; ROC, receiver operating characteristic; SVM, Support Vector Machine.

According to the results of Table 3, among the models, the DT model with an accuracy value of 0.75 has the highest accuracy among the 3 models. Also, the DT model with a sensitivity of 0.60 had the highest sensitivity among the models. For the two‐feature specificity and Area under the ROC curve, the DT obtained the highest values with values of 0.68 and 0.71 respectively. After the DT model, the AdaBoost model ranks second with values of accuracy, sensitivity, specificity, and area under the ROC curve of 0.74, 0.58, 0.65, and 0.68 respectively. The SVM model is placed in the last place with values of accuracy, sensitivity, specificity, and area under the ROC curve of 0.70, 0.54, 0.62, and 0.63, which shows poor performance compared to the other two models.

## 4 DISCUSSION

Death due to covid‐19 is one of the biggest health challenges in the world. Predicting the death of covid‐19 patients can be effective in the allocation of human resources and treatment resources. There are many models that can predict death due to COVID‐19. DT, SVM, and Adabost models were separately fitted for death caused by COVID‐19.^25^, ^26^, ^27^ But these three models have not been compared at the same time. Therefore, this issue is the strength of the present study. The results of the present study showed that the accuracy of DT model was higher than SVM and AdaBoost. The results of the study of Parsapour et al.^28^ in predicting the severity of depression disorder, the study of NateghiNia et al.^29^ in predicting the condition of patients staying in the intensive care unit, the study of Nazeri et al.^30^ in the prediction of breast cancer metastasis, the study of Heidari et al.^31^ in the diagnosis of infertility showed that the accuracy and predictive power of DT was higher than K‐means, neural network, SVM, and K‐nearest neighbor. The results of these studies were consistent with the results of the present study. It seems that DT has a high predictive power compared to other data mining models in different data sets. Therefore, it is suggested to researchers in different fields to use DT to predict the studied variables. Also, considering that in this study the Decision Tree (DT) had 75% accuracy, it is suggested to use other approaches such as random forest or XGBoost to improve the DT in future studies.

The results of statistical tests in the present study showed that there was a significant relationship between gender, place of hospitalization, how to refer, contact history, fever, frequent coughs, severe muscle pains, stress and mental distress, low level of consciousness, loss of smell, loss of taste, abdominal pain, nausea, vomit, diarrhea, unprecedented anorexia, dizziness, chest pain, intubation, various types of cancer, chemotherapy, diabetes, chronic blood disease, defects in the body's immune system, pregnancy, dialysis, chronic lung disease and the death of patients with covid‐19. The results of the study of Kouhpeikar et al.^8^ the study of Kouhpeikar et al.^32^ the study of Naghibifar et al.^2^ were in line with the results of the present study. Therefore, choosing these variables as predictive variables increases the value and accuracy of the models.

Other one of notable strength of the current study lies in the utilization of an extensive and comprehensive data set, encompassing a population of 23,504 COVID‐19 patients. Furthermore, this data set was meticulously gathered from every hospital and health center within the city of Kermanshah, located in Western Iran. The findings of this study need to be interpreted in light of certain limitations. First, as an observational study, potential unmeasured intervening variables may have influenced the results. Second, like any observational study, there is a risk of data bias, particularly in self‐reported variables. Third, it is important to exercise caution when generalizing the results to other ethnicities, as the majority of the population in Kermanshah is of Kurdish ethnicity.

## AUTHOR CONTRIBUTIONS

All authors have read and approved the final version of the manuscript. Bita Shokri Gharehhasani had full access to all of the data in this study and takes complete responsibility for the integrity of the data and the accuracy of the data analysis.

## CONFLICT OF INTEREST STATEMENT

The authors declare no conflict of interest.

## ETHICS STATEMENT

This research has been approved by the code of ethics IR.KUMS.REC.1400.741 in Kermanshah University of Medical Sciences.

## TRANSPARENCY STATEMENT

The lead author Bita Shokri gharehhasani affirms that this manuscript is an honest, accurate, and transparent account of the study being reported; that no important aspects of the study have been omitted; and that any discrepancies from the study as planned (and, if relevant, registered) have been explained.

## Data Availability

The data that support the findings of this study are available from the corresponding author upon reasonable request. The data sets generated and/or analyzed during the current study are not publicly available to protect the confidentiality of the participants.
